# Preoxygenation with standard facemask combining apnoeic oxygenation using high flow nasal cannula versuss standard facemask alone in patients with and without obesity: the OPTIMASK international study

**DOI:** 10.1186/s13613-023-01124-x

**Published:** 2023-04-04

**Authors:** Samir Jaber, Audrey De Jong, Maximilian S. Schaefer, Jiaqiang Zhang, Xiaowen Ma, Xinrui Hao, Shujing Zhou, Shang Lv, Valerie Banner-Goodspeed, Xiuhua Niu, Thomas Sfara, Daniel Talmor

**Affiliations:** 1 Anesthesiology and Intensive Care; Anesthesia and Critical Care Department B, Saint Eloi Teaching Hospital, PhyMedExp, University of Montpellier, INSERM U1046, 1; 80 avenue Augustin Fliche, Montpellier cedex 5, Montpellier, France; 2 Centre Hospitalier Universitaire Montpellier, 34295 Montpellier, France; 3 Center for Anesthesia Research Exellence, Department of Anesthesia, Critical Care and Pain Medicine, Beth Israel Deaconess Medical Center and Harvard Medical School, Boston, USA; 4 Department of Anesthesiology and Perioperative Medicine, People’s Hospital of Zhengzhou University, Henan Provincial People’s Hospital, Zhengzhou, Henan China; 5 Department of Anesthesiology, Renji Hospital, Shanghai Jiaotong University, School of Medicine, Shanghai, China; 6 Shenzhen Mindray Bio-Medical Electronics Co., Ltd. Mindray Building, Keji 12th Road South, High-tech Industrial Park, Nanshan, Shenzhen, 518057 People’s Republic of China

**Keywords:** Intubation, Obesity, Operating room, Preoxygenation, Videolaryngoscope, Anaesthesia

## Abstract

**Background:**

Combining oxygen facemask with apnoeic oxygenation using high-flow-nasal-oxygen (HFNO) for preoxygenation in the operating room has not been studied against standard oxygen facemask alone. We hypothesized that facemask-alone would be associated with lower levels of lowest end-tidal oxygen (EtO2) within 2 min after intubation in comparison with facemask combined with HFNO.

**Methods:**

In an international prospective before–after multicentre study, we included adult patients intubated in the operating room from September 2022 to December 2022. In the before period, preoxygenation was performed with facemask-alone, which was removed during laryngoscopy. In the after period, facemask combined with HFNO was used for preoxygenation and HFNO for apnoeic oxygenation during laryngoscopy. HFNO was maintained throughout intubation. The primary outcome was the lowest EtO2 within 2 min after intubation. The secondary outcome was SpO2 ≤ 95% within 2 min after intubation. Subgroup analyses were performed in patients without and with obesity. This study was registered 10 August 2022 with ClinicalTrials.gov, number NCT05495841.

**Results:**

A total of 450 intubations were evaluated, 233 with facemask-alone and 217 with facemask combined with HFNO. In all patients, the lowest EtO2 within 2 min after intubation was significantly lower with facemask-alone than with facemask combined with HFNO, 89 (85–92)% vs 91 (88–93)%, respectively (mean difference − 2.20(− 3.21 to − 1.18), *p* < 0.001). In patients with obesity, similar results were found [87(82–91)% vs 90(88–92)%, *p* = 0.004]; as in patients without obesity [90(86–92)% vs 91(89–93)%, *p* = 0.001)]. SpO2 ≤ 95% was more frequent with facemask-alone (14/232, 6%) than with facemask combined with HFNO (2/215, 1%, *p* = 0.004). No severe adverse events were recorded.

**Conclusions:**

Combining facemask with HFNO for preoxygenation and apnoeic oxygenation was associated with increased levels of lowest EtO2 within 2 min after intubation and less desaturation.

**Supplementary Information:**

The online version contains supplementary material available at 10.1186/s13613-023-01124-x.

## Background

Invasive mechanical ventilation is needed for most surgical procedures with general anaesthesia in the operative room. This requires tracheal intubation, which is one of the most frequent procedures performed in the operating room. Risks associated with tracheal intubation include severe hypoxemia during the procedure that can result in cardiac arrest, cerebral anoxia and death [1].

Preoxygenation using administration of 100% oxygen before the induction of general anaesthesia enhances oxygen reserves and delays hypoxemia [2]. Preoxygenation is especially important if difficult airway management is anticipated or in patients at high risk of desaturation [3]. Patients with obesity are more likely to desaturate than are lean patients [4]. Maximal oxygen administration through preoxygenation is a major component of safety for these patients during induction of general anaesthesia [5]. The usual technique of oxygen administration is for the patient to spontaneously breathe 100% oxygen by facemask for 3–5 min.[6]

A new method of oxygenation, high-flow nasal oxygen (HFNO), has been introduced in the operating room [7], which delivers high flow, heated and humidified gas via nasal cannula at a prescribed fraction of inspired oxygen (FiO2) and a maximum flow greater than 60 L/min. HFNO is maintained throughout the intubation procedure, whereas a facemask is removed when apnea occurs to allow laryngoscopy. HFNO is, therefore, both a method for preoxygenation and apnoeic oxygenation.

Recent studies suggest that HFNO allows for apnoeic oxygenation and interestingly as a consequence could be used to improve blood oxygenation during the apnoeic period of intubation, when the facial mask is removed. This may be particularly useful in obese patients [8–11].

Data are lacking regarding the effect on oxygenation of combining facemask preoxygenation and apnoeic oxygenation using HFNO in operating room. Lowest end-tidal oxygen concentration (EtO2) within the 2 min after intubation is a criterion often used as a surrogate of oxygen reserves [12].

To our knowledge, no study has compared the lowest EtO2 within the 2 min after intubation using standard facemask preoxygenation vs facemask preoxygenation combined with HFNO for both preoxygenation and apnoeic oxygenation in unselected patients.

We hypothesized that combining facemask preoxygenation with HFNO for preoxygenation and apnoeic oxygenation would be associated with increased levels of lowest EtO2 within 2 min after intubation, both in overall patients and in patients without and with obesity.

## Methods

### Study design and setting

**Fig. 1:**
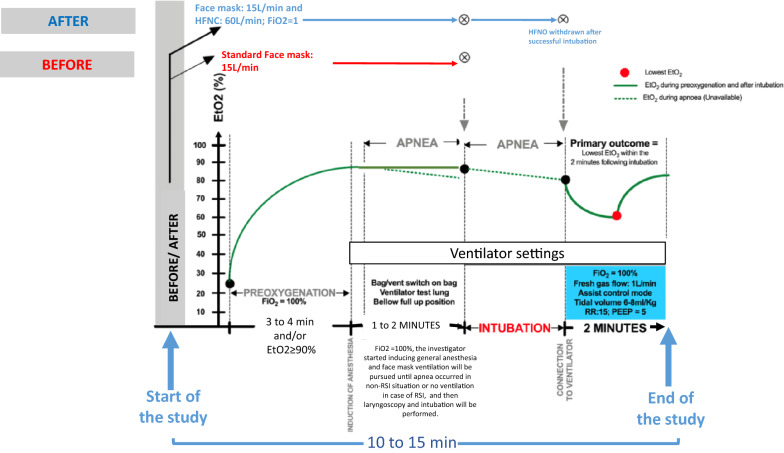
Study design. *EtO2* end-tidal oxygen, *PEEP* positive end expiratory pressure, *HFNO* High-Flow Nasal cannula Oxygen, *RR* respiratory rate, *RSI* rapid sequence induction

An international, before and after, prospective study was performed. The study design is detailed in Fig. 1. Preoxygenation was performed during 3–4 min and/or EtO2 ≥ 90%. During 1–2 min following induction of general anaesthesia, the patient received 100% oxygen and face mask ventilation was pursued until apnea occurred in non-rapid sequence induction (RSI) situation or no ventilation was performed in case of RSI. Then, laryngoscopy and intubation were performed. In the before period, standard oxygen facemask preoxygenation was used (Additional file 1: Fig. S1A). In the after period, combination of standard oxygen facemask preoxygenation with HFNO for both preoxygenation and apnoeic oxygenation was used (Additional file 1: Fig. S1B). For both periods, a Mindray anaesthesia workstation ventilator A8 or A9 (Mindray Medical, Shenzhen, Guangdong, China) was the ventilator, which allows both facemask ventilation and HFNO through an auxiliary mounted flowmeter graduated to 60 l/min on the same device.

An attending anaesthesiologist, a resident anaesthesiologist or a certified registered nurse anaesthesiologist performed tracheal intubation. The anaesthesia technique, including choice of drugs, conduction of rapid sequence induction and use of airway devices, was at the discretion of the anaesthesia provider.

### Patients and ethics

The appropriate ethical approval was obtained at each participating institution and the study conducted in accordance with the principles of the Declaration of Helsinki. The need for informed consent was waived at three centres on the basis that the study did not require any research procedures other than passive data collection, while one centre required prospective informed consent from participants. The final database was de-identified.

The Strengthening the Reporting of OBservational Studies in Epidemiology checklist was used in preparing this manuscript. The study was prospectively registered 10 August 2022 on clinicaltrials.gov as NCT05495841 prior to inclusion of the first participant.

Four centres participated, Boston (USA), Zhengzhou (China), Shanghai (China) and Montpellier (France) (Additional file 1: Table S1). From September 2022 to December 2022, adult patients (18 years or older) for scheduled or non-scheduled surgery with or without indication of rapid sequence induction (full stomach) undergoing general anaesthesia with tracheal intubation were included. Inclusions were consecutively performed in each centre when an appropriate anaesthesia machine was available in the operating room as well as the research team.

Exclusion criteria were age < 18 years, hemodynamic instability, intubation without laryngoscopy (i.e., fibreoptic intubation for anticipated “cannot ventilate situation” or mouth opening < 2 cm), facial surgery, adults subject to legal protection and known pregnancy (due to higher risk of oxygen desaturation and aspiration).

### Data source and data collection

Members of the research team prospectively recorded the data in a standardized manner.

Baseline characteristics were assessed: age, gender, body mass index (BMI, the weight in kilograms divided by the square of the height in meters), Obesity (defined as BMI ≥ 30 kg/m^2^), American Society of Anaesthesiology (ASA) Score, Mallampati score, Peripheral oxygen saturation (SpO2) before intubation, systolic and diastolic blood pressures before intubation, heart rate and respiratory rate before intubation; and anaesthesia and surgery characteristics: type of surgery, drugs of anaesthesia used for induction (hypnotic, opioid and neuromuscular blockers), rapid sequence induction use, device used for intubation (Macintosh laryngoscope or videolaryngoscope). Severe adverse events were defined as death and cardiac arrest during intubation.

### Outcomes

The primary outcome was the lowest EtO2 value within the 2 min after tracheal intubation [12].

Secondary outcomes were the lowest SpO2 value during intubation and within the 2 min after intubation, the lowest and highest levels of end-tidal carbon dioxide concentration (EtCO2) within the 2 mins after intubation, the SpO2 value at the end of preoxygenation, the first EtO2 and the first SpO2 after intubation, the occurrence of desaturation defined by SpO2 ≤ 95% during intubation and within 2 min after intubation, the tolerance of the device (discomfort considered if the patient asks for reduction of the gas flow of HFNO, and subjective assessment of ease of preoxygenation assessed by the operator on a numeric scale from 0 to 10), the first-attempt success before successful tracheal intubation and the subjective assessment of excellent conditions of intubation. First-attempt intubation success was defined as tracheal tube placement (confirmed by persistent EtCO2) with a single blade insertion and without manipulation of the laryngoscope by another provider [6].

### Statistical analysis

Statistical analysis was performed using SAS software version 9.4 (SAS Institute, Cary, NC) and R software (version 3.0.2). A data analysis and statistical plan was written before the data were accessed. To demonstrate a difference in the lowest EtO2 within the 2 min after intubation of 5% (standard deviation of 12%), from 85% with facemask alone (before period study) to 90% with combined facemask and HFNO (after period study), 150 tracheal intubations per period (total of 300 tracheal intubations) were required, with a power of 95% and an alpha risk of 5%. To compensate for dropouts and missing data, we decided to include 450 tracheal intubations.

Quantitative data were expressed as median and interquartile range (IQR) and compared using Student’s *t* test if normally distributed and the Mann–Whitney test if not normally distributed. Qualitative data were expressed as number (percentage) and compared using the Chi-square test. No imputation was made for missing data, and analyses were made on complete cases.

Outcomes were assessed first for the overall population and subsequently in patients with and without obesity.

Primary outcome (lowest EtO2 within 2 min after intubation) was compared between the facemask group and the facemask with HFNO group using a Wilcoxon test (unadjusted analysis). Interaction between groups and obesity were searched. A post hoc analysis was performed in the subgroup of patients with rapid sequence induction.

Comparisons of secondary outcomes were performed using Chi square tests (or Fisher tests when appropriate) for qualitative data and the Student’s *t* tests (or Wilcoxon tests when appropriate) for quantitative data. For qualitative data, relative risk and 95% confidence interval were estimated with the Mantel–Haenszel method. No adjustment was made based on the multiplicity.

The absolute difference, relative risk and their 95% confidence interval (CI) were calculated.

Then, to take into account baseline characteristics differences between the groups, a multivariable mixed generalized linear model taking into account the centre as a random effect was performed to provide adjusted results of lowest EtO2 within 2 min after intubation (primary outcome), considering a priori that Mallampati score, age, sex, obesity, ASA score, type of surgery, videolaryngoscope use and rapid sequence induction would be confounding factors. These factors were entered into the multivariable model, and a final model including only significant variables was computed. A similar multivariable mixed generalized logistic model was performed for the occurrence of desaturation during and within 2 min after intubation (SpO2 ≤ 95%). Following these multivariable models, odds ratio with 95% CI were computed.

All tests were two-tailed and *p* values of less than 0.05 were considered significant.

## Results

### Baseline characteristics

A total of 450 intubations were performed, 233 in the before period with facemask alone (48 with obesity and 185 without obesity) and 217 in the after period with facemask combined with HFNO (45 with obesity and 172 without obesity) (Additional file 1: Table S1 for details by centre).

Demographic and tracheal intubation characteristics according to study group are summarized in Table 1. There were no significant differences between groups.

**Table 1:** Baseline characteristics of the overall participants

	Overall (*n* = 450)	Facemask alone (*n* = 233)	Facemask combined with HFNO (*n* = 217)
Age (years)	57 (47–68)	59 (48–67)	62 (45–68)
Female sex	218/449 (48.6)	119 (51.1)	99/216 (45.8)
Body-mass index^a^	25 (23–29)	25 (22–29)	25 (23–29)
Obesity^b^	93 (20.7)	48 (20.6)	45 (20.7)
ASA score 1 or 2	325 (72.2)	167 (71.7)	158 (72.8)
Mallampati score 1 or 2	370/445 (83.1)	195/231 (84.4)	175/214 (81.7)
Type of surgery			
Abdominal	214 (47.6)	110 (47.0)	104 (48.0)
Cardiovascular	25 (5.6)	12 (5.2)	13 (6.0)
Neurosurgery	21 (4.7)	13 (5.6)	8 (3.7)
Orthopaedic	14 (3.1)	4 (1.7)	10 (4.6)
Gynaecologic	32 (7.1)	22 (9.4)	10 (4.6)
Thoracic	17 (3.8)	11 (4.7)	17 (3.8)
Otolaryngology	21 (4.7)	21 (4.7)	6 (2.8)
Urologic	90 (20.0)	45 (19.0)	45 (21.0)
Plastic	9 (2.0)	5 (2.1)	4 (1.8)
Endoscopy	2 (0.4)	2 (0.9)	0 (0)
Other	5 (1.1)	2 (0.9)	3 (1.4)
SpO2 (%)^c^	98 (97–99)	98 (97–99)	98 (97–99)
Systolic blood pressure (mmHg)^d^	137 (124–152)	136 (122–151)	139 (126–154)
Diastolic blood pressure (mmHg)^d^	81 (72–89)	81 (72–87)	81 (73–89)
Heart rate (/min)	76 (67–85)	77 (68–84)	74 (66–87)
Respiratory rate (/min)^e^	16 (13–18)	16 (14–18)	15 (13–18)
Hypnotic used for induction			
Propofol	309/449 (68.8)	159/232 (68.5)	150 (69.1)
Etomidate	139/449 (31.0)	72/232 (31.1)	67 (30.9)
Ketamine	1/449 (0.2)	1/232 (0.4)	0 (0.0)
Opioid used for induction			
None	42 (9.3)	23 (9.9)	19 (8.8)
Sufentanil	303 (67.3)	151 (65.0)	152 (70.0)
Remifentanil	4 (0.9)	3 (1.3)	1 (0.5)
Fentanyl	97 (21.6)	55 (24.0)	42 (19.0)
Hydromorphone	4 (0.9)	1 (0.4)	3 (1.4)
Neuromuscular blocker used for induction			
None	1 (0.2)	1 (0.4)	0 (0)
Rocuronium	364 (80.9)	187 (80.3)	177 (81.6)
Atracurium	1 (0.2)	0 (0)	1 (0.5)
Cisatracurium	62 (13.8)	33 (14.2)	29 (13.4)
Succinylcholine	22 (4.9)	12 (5.2)	10 (4.6)
Rapid sequence induction	73 (16.2)	33 (14.2)	40 (18.4)
Intubation device			
Videolaryngoscope	334 (74.2)	164 (70.4)	170 (78.3)
Macintosh laryngoscope	116 (25.8)	69 (29.6)	47 (21.7)

Data are *n*/*N* (%), and median (IQR)

*HFNO* High-Flow Nasal cannula Oxygen, *ASA* American Society of Anaesthesiology, *NA* not applicable

^a^Body Mass Index is the weight in kilograms divided by the square of the height in meters

^b^Obesity was defined as body mass index ≥ 30 kg/m^2^

^c^Data on SpO2 were missing for 0 patients (0%) in the facemask alone group and 1 (0.46%) in the facemask combined with HFNO group

^d^Data on systolic and diastolic blood pressure were missing for 0 patients (0%) in the facemask alone group and 1 (0.46%) in the facemask combined with HFNO group

^e^Data on respiratory rate were missing for 0 patients (0%) in the facemask alone group and 4 (1.8%) in the facemask combined with HFNO group

### Primary outcome: lowest EtO2 within 2 min after intubation

**Fig. 2:**
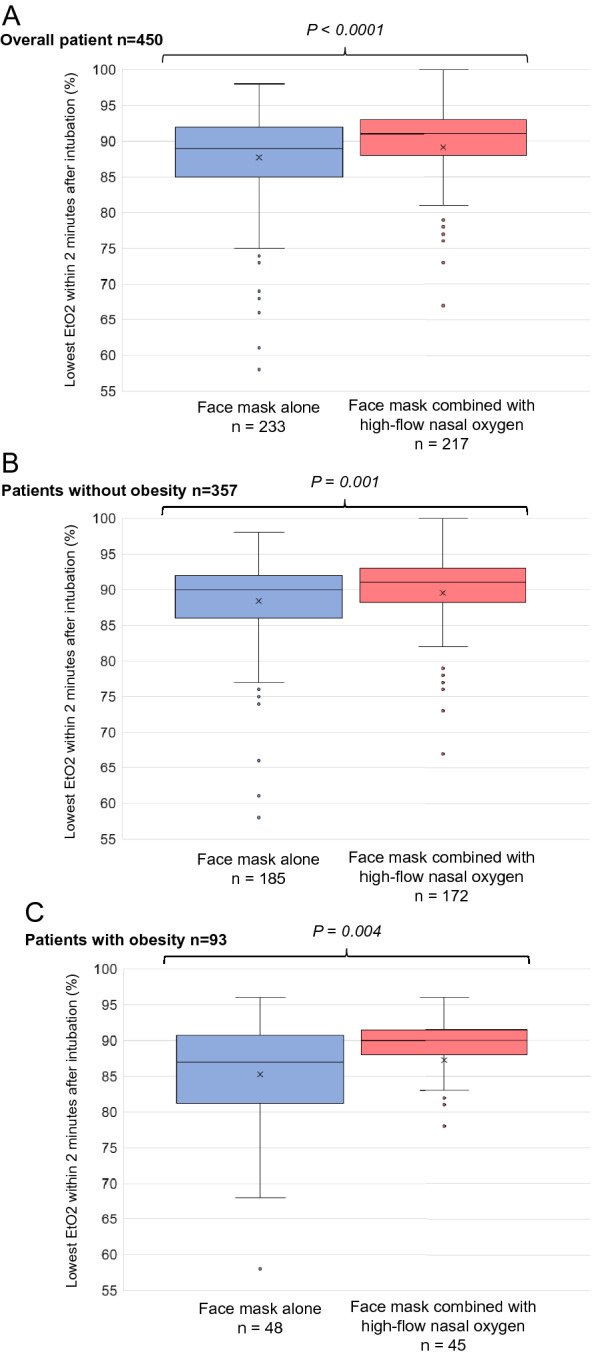
Primary outcome: end-tidal oxygen (EtO2) levels within 2 min after intubation in the facemask alone group and the facemask combined with HFNO group, and according to obesity status **A**. In overall patients **B**. In patients without obesity **C**. In patients with obesity *EtO2* End-tidal oxygen

The lowest EtO2 within the 2 min after intubation was significantly lower with facemask alone than with facemask combined with HFNO, 89 (85–92)% vs 91 (88–93)%, respectively (mean difference − 2.20 (− 3.21 to − 1.18), *p* < 0.001, Table 2, Fig. 2). Baseline characteristics of patients with obesity are presented in Table 3 and were clinically similar between groups. Interaction between group and obesity was not significant. In patients with obesity, the lowest EtO2 within the 2 min after intubation was significantly lower with facemask alone than with facemask combined with HFNO [(87 (82–91)% vs 90 (88–92)%, mean difference − 4.02 (− 6.58 to − 1.47), *p* = 0.004), which was also the case in patients without obesity (90 (86–92)% vs 91 (89–93)%, mean difference − 1.72 (− 2.81 to − 0.63), *p* = 0.001), Table 4, Fig. 2).

**Table 2:** Outcomes

	Overall (*n* = 450)	Facemask alone (*n* = 233)	Facemask combined with HFNO (*n* = 217)	Absolute differences (95% CI)	Relative risks (95% CI)	*p* value
Primary: lowest EtO2 within 2 min after intubation (%)^a^	90 (87–92)	89 (85–92)	91 (88–93)	− 2.20 (− 3.21 to − 1.18)	–	< 0.001
Secondary:						
SpO2 at the end of preoxygenation (%)	100 (100–100)	100 (100–100)	100 (100–100)	0.02 (− 0.08 to 0.12)		0.56
First EtO2 after intubation (%)^b^	91 (88–96)	90 (87–96)	92 (89–97)	− 1.64 (− 2.83 to − 0.45)	–	0.004
Highest EtCO2 after intubation (%)^c^	37 (34–40)	37 (34–40)	37 (33–41)	0.83 (− 0.21 to 1.87)	–	0.26
Lowest EtCO2 within 2 min after intubation (%)^c^	35 (32–38)	35 (32–38)	35 (32–38)	0.76 (− 0.22 to 1.73)	–	0.38
First SpO2 after intubation (%)^d^	100 (100–100)	100 (100–100)	100 (100–100)	− 0.56 (− 1.17 to 0.05)	–	0.39
Lowest SpO2 during intubation and within 2 min after intubation (%)^d^	100 (99–100)	100 (99–100)	100 (99–100)	− 0.64 (− 1.36 to 0.09)	–	0.41
SpO2 ≤ 95% during intubation and within 2 min after intubation	16/447 (3.6)	14/232 (6.0)	2/215 (0.93)	0.05 (0.02 to 0.09)	6.84 (1.54 to 30.5)	0.004
Ease of use of preoxygenation (from 0 to 10)^e^	9 (8–10)	10 (9–10)	8 (7–9)	1.74 (1.36 to 2.11)	–	< 0.001
First-attempt success	416/441 (94.3)	210/225 (93.3)	206/216 (93.4)	− 0.02 (− 0.06 to 0.02)	0.68 (0.30 to 1.55)	0.36
Excellent conditions of intubation	253/406 (62.3)	127/209 (60.8)	126/197 (64.0)	− 0.03 (− 0.13 to 0.06)	0.87 (0.58 to 1.31)	0.51

Data are *n*/*N* (%), and median (IQR)

*SpO2* Peripheral oxygen saturation, *EtO2* end-tidal oxygen, *EtCO2* end-tidal carbon dioxide, *HFNO* High-Flow Nasal cannula Oxygen, *NA* Not Applicable

^a^Data on lowest EtO2 after intubation were missing for 0 patients (0%) in the facemask alone group and 2 (1.0%) in the facemask combined with HFNO group

^b^Data on first EtO2 after intubation were missing for 9 patients (3.9%) in the facemask alone group and 4 (1.8%) in the facemask combined with HFNO group

^c^Data on highest and lowest EtCO2 after intubation were missing for 0 patients (0%) in the facemask alone group and 2 (1.0%) in the facemask combined with HFNO group

^d^Data on first and lowest SpO2 after intubation were missing for 0 patients (0%) in the facemask alone group and 2 (1.0%) in the facemask combined with HFNO group

^e^Data on ease of use of preoxygenation were missing for 73 patients (31.3%) in the facemask alone group and 49 (22.6%) in the facemask combined with HFNO group

**Table 3:** Baseline characteristics according to obesity status

	Patients without obesity	Facemask alone (*n* = 185)	Facemask combined with HFNO (*n* = 172)	Patients with obesity	Facemask alone (*n* = 48)	Facemask combined with HFNO (*n* = 45)
	Overall (*n* = 357)			Overall (*n* = 93)		
Age (years)	62 (49–69)	60 (49–69)	63 (49–69)	54 (38–65)	55 (38–64)	54 (38–65)
Female sex	157/356 (44.1)	85 (45.9)	72/171 (42.1)	61 (65.6)	34 (70.8)	27 (60.0)
Body-mass index^a^	24 (22–26)	24 (22–26)	24 (22–26)	35 (32–40)	34 (32–38)	36 (32–42)
ASA score 1 or 2	264 (74.0)	135 (73.0)	129 (75.0)	61 (65.6)	32 (66.7)	29 (64.4)
Mallampati score 1 or 2	307/354 (86.7)	160/183 (87.4)	147/171 (86.0)	63 (69.2)	35 (72.9)	28/43 (65.1)
Type of surgery						
Abdominal	155 (43.4)	80 (43.2)	75 (43.6)	59 (63.4)	30 (62.5)	29 (64.4)
Cardiovascular	22 (6.2)	11 (5.9)	11 (6.4)	3 (3.2)	1 (2.1)	2 (4.4)
Neurosurgery	17 (4.8)	10 (5.4)	7 (4.1)	4 (4.3)	3 (6.3)	1 (2.2)
Orthopaedic	10 (2.8)	2 (1.1)	8 (4.7)	4 (4.3)	2 (4.2)	2 (4.4)
Gynaecologic	25 (7.0)	18 (9.7)	7 (4.1)	7 (7.5)	4 (8.3)	3 (6.7)
Thoracic	12 (3.4)	7 (3.8)	5 (2.9)	5 (5.4)	4 (8.3)	1 (2.2)
Otolaryngology	18 (5.0)	7 (3.8)	11 (6.4)	3 (3.2)	0 (0)	3 (6.7)
Urologic	87 (24.4)	44 (23.8)	43 (25.0)	3 (3.2)	1 (2.1)	2 (4.4)
Plastic	5 (1.4)	3 (1.6)	2 (1.2)	4 (4.3)	2 (4.2)	2 (4.4)
Endoscopy	2 (0.6)	2 (1.1)	0 (0)	0 (0)	0 (0)	0 (0)
Other	4 (1.1)	1 (0.54)	3 (1.7)	1 (1.1)	1 (2.1)	0 (0)
SpO2 (%)^b^	98 (97–99)	98 (97–99)	98 (97–99)	98 (96–99)	98 (97–99)	98 (96–99)
Systolic blood pressure (mmHg)^c^	139 (126–154)	137 (124–153)	140 (127–157)	132 (118–148)	129 (114–150)	138 (122–146)
Diastolic blood pressure (mmHg)^c^	81 (73–89)	81 (72–88)	82 (74–90)	79 (71–86)	81 (72–87)	77 (71–85)
Heart rate (/min)	74 (66–84)	76 (67–84)	73 (65–85)	78 (69–88)	79 (70–88)	78 (67–88)
Respiratory rate (/min)^d^	16 (13–18)	16 (14–18)	15 (13–18)	16 (14–18)	16 (14–18)	16 (14–17)
Hypnotic used for induction						
Propofol	225/356 (63.2)	115/184 (62.5)	110 (64.0)	84 (90.3)	44 (91.7)	40 (88.9)
Etomidate	131/356 (36.8)	69/184 (37.5)	62 (36.0)	8 (8.6)	3 (6.3)	5 (11.1)
Ketamine	0 (0)	0 (0)	0 (0)	1 (1.1)	1 (2.1)	0 (0)
Opioid used for induction						
None	22 (6.2)	13 (7.0)	9 (5.2)	20 (21.5)	10 (20.9)	10 (22.2)
Sufentanil	263 (73.7)	132 (71.4)	131 (76.2)	40 (43.0)	19 (39.6)	21 (46.7)
Remifentanil	3 (0.84)	3 (1.6)	0 (0)	1 (1.1)	0 (0)	1 (2.2)
Fentanyl	65 (18.2)	36 (19.5)	29 (16.9)	32 (34.4)	19 (39.6)	13 (28.9)
Hydromorphone	4 (1.1)	1 (0.54)	3 (1.7)	0 (0)	0 (0)	0 (0)
Neuromuscular blocker used for induction						
None	1 (0.28)	1 (0.4)	0 (0)	0 (0)	0 (0)	0 (0)
Rocuronium	289 (90.0)	150 (81.1)	139 (80.9)	75 (80.7)	37 (77.1)	38 (84.4)
Atracurium	1 (0.28)	0 (0)	1 (0.58)	0 (0)	0 (0)	0 (0)
Cisatracurium	51 (14.3)	27 (14.6)	24 (14.0)	11 (11.8)	6 (12.5)	5 (11.1)
Succinylcholine	15 (4.2)	7 (3.8)	8 (4.7)	7 (7.5)	5 (10.4)	2 (4.4)
Rapid sequence induction	32 (9.0)	15 (8.1)	17 (9.9)	41 (44.1)	18 (37.5)	23 (51.1)
Intubation device						
Videolaryngoscope	265 (74.2)	128 (69.2)	137 (79.7)	69 (74.2)	36 (75.0)	33 (73.3)
Macintosh laryngoscope	92 (25.8)	57 (30.8)	35 (20.3)	24 (25.8)	12 (25.0)	12 (26.7)

Data are *n*/*N* (%), and median (IQR)

*HFNO* High-Flow Nasal cannula Oxygen, *ASA* American Society of Anaesthesiology, *NA* not applicable

^a^Body Mass Index is the weight in kilograms divided by the square of the height in meters

^b^Data on SpO2 before intubation were missing for 0 patients (0%) in patients with obesity and 1 (0.28%) in patients without obesity

^c^Data on systolic and diastolic blood pressure before intubation were missing for 0 patients (0%) in patients with obesity and 1 (0.28%) in patients without obesity

^d^Data on respiratory rate before intubation were missing for 1 patients (1.1%) in patients with obesity and 3 (0.84%) in patients without obesity

**Table 4:** Outcomes according to the obesity status

	Patients without obesity	Facemask alone (*n* = 185)	Facemask combined with HFNO (*n* = 172)	*p* value	Patients with obesity	Facemask alone (*n* = 48)	Facemask combined with HFNO (*n* = 45)	*p* value
	Overall (*n* = 357)				Overall (*n* = 93)			
Primary: lowest EtO2 within 2 min after intubation (%)^a^	90 (88–93)	90 (86–92)	91 (89–93)	0.001	89 (85–91)	87 (82–91)	90 (88–92)	0.004
Secondary:								
SpO2 at the end of preoxygenation (%)	100 (100–100)	100 (100–100)	100 (100–100)	0.18	100 (100–100)	100 (100–100)	100 (100–100)	0.28
First EtO2 after intubation (%)^b^	91 (88–100)	91 (87–100)	92 (89–99)	0.05	90 (86–92)	89 (86–92)	90 (89–94)	0.01
Highest EtCO2 after intubation (%)^b^	37 (34–41)	37 (34–40)	37 (33–41)	0.28	37 (34–40)	37 (34–40)	36 (34–39)	0.68
Lowest EtCO2 within 2 min after intubation (%)^c^	35 (32–38)	35 (32–38)	35 (33–39)	0.60	36 (32–38)	36 (32–39)	35 (32–37)	0.34
First SpO2 after intubation (%)^d^	100 (100–100)	100 (100–100)	100 (100–100)	0.43	100 (99–100)	100 (99–100)	100 (99–100)	0.76
Lowest SpO2 during intubation and within 2 min after intubation (%)^d^	100 (99–100)	100 (100–100)	100 (99–100)	0.52	100 (98–100)	100 (98–100)	100 (98–100)	0.76
SpO2 ≤ 95% during intubation and within 2 min after intubation	7/356 (2.0)	6/185 (3.2)	1/171 (0.58)	0.12	9/91 (9.9)	8/47 (17.0)	1/44 (2.3)	0.03
Ease of use of preoxygenation (from 0 to 10)^e^	9 (8–10)	10 (9–10)	8 (7–9)	< 0.001	9 (7–10)	10 (9–10)	8 (6–9)	< 0.001
First-attempt success	333/352 (94.6)	169/181 (93.4)	164/171 (95.9)	0.29	83/89 (93.3)	41/44 (93.2)	42/45 (93.3)	1.00
Excellent conditions of intubation	202/326 (62.0)	103/169 (60.9)	99/157 (63.1)	0.69	51/80 (63.8)	24/40 (60.0)	27/40 (67.5)	0.49

Data are *n*/*N* (%), and median (IQR)

*SpO2* Peripheral oxygen saturation, *EtO2* end-tidal oxygen, *EtCO2* end-tidal carbon dioxide, *HFNO* High-Flow Nasal cannula Oxygen

^a^Data on lowest EtO2 after intubation were missing for 1 patients (1.0%) in patients with obesity and 1 (0.3%) in patients without obesity

^b^Data on first EtO2 after intubation were missing for 2 patients (2.2%) in patients with obesity and 11 (3.1%) in patients without obesity

^c^Data on highest and lowest EtCO2 after intubation were missing for 1 patients (1.0%) in patients with obesity and 1 (0.3%) in patients without obesity

^d^Data on first and lowest SpO2 after intubation were missing for 1 patients (1.0%) in patients with obesity and 1 (0.3%) in patients without obesity

^e^Data on ease of use of preoxygenation were missing for 40 patients (43.0%) in patients with obesity and 82 (23.0%) in patients without obesity

In the 73 patients with RSI, the lowest EtO2 within the 2 min after intubation was significantly lower with facemask alone than with facemask combined with HFNO, 86 (81–88)% vs 89 (86–91)%, respectively, *p* = 0.005).

After multivariate analysis, receiving facemask alone and having a Mallampati score of 3 or 4 were independently associated with lowest EtO2 within 2 min after intubation (*p* < 0.0001 and *p* = 0.04, respectively).

### Secondary outcomes

Secondary outcomes are presented in Table 2 for the overall population and in Table 4 for patients with and without obesity.

A SpO2 ≤ 95% was more frequent with facemask alone (14/232, 6%) than with facemask combined with HFNO (2/215, 1%, *p* = 0.004), especially in patients with obesity (8/47 (17.0) vs 1/44 (2.3), *p* = 0.03, Table 4, Additional file 1: Fig. S2).

In multivariable analyses, receiving facemask alone and having obesity were independently associated with SpO2 ≤ 95% [OR = 7.16 95% CI (1.58–32.4)], *p* = 0.01 and OR = 5.46 95% CI (1.91–15.7), *p* = 0.0016, respectively).

First EtO2 after intubation was significantly lower with facemask alone than with facemask combined with HFNO (Table 2).

Facemask alone was better tolerated than facemask combined with HFNO as suggested by higher scores of ease of use of preoxygenation, and frequent patient request to reduce gas flow in the facemask combined with HFNO group [61/168 (36.3%)] (Table 2).

No significant differences were found for other outcomes. No severe adverse events were recorded.

## Discussion

In this international prospective multicentre before after study performed in 450 tracheal intubation procedures, the use of facemask combined with HFNO for both preoxygenation and apnoeic oxygenation in unselected patients in the operating room was associated with an increase in the lowest EtO2 within the 2 min after intubation and a lower rate of oxygen desaturation, when compared with preoxygenation with facemask alone.

To our knowledge, this is the first study to assess if the implementation of the use of facemask combined with HFNO for both preoxygenation and apnoeic oxygenation in real-life operating rooms improved the EtO2 levels following intubation.

EtO2 at the end of preoxygenation is a reliable indicator of the alveolar partial pressure (PAO2) and is the gold standard of monitoring during anaesthesia in international guidelines [6, 13]. During pre-oxygenation, the commonly adopted objective of EtO2 is 90% [2, 14]. The benefit of maximum pre-oxygenation (FiO2 = 100%) is greater than the risk of developing atelectasis [15].

Clinical studies have evaluated the effect of preoxygenation and apnoeic oxygenation using HFNO, with conflicting results [16]. The apparent contradictory results of these studies could be explained in part by differences in study design, oxygen therapy settings and patient characteristics. The effectiveness of apnoeic oxygenation depends on the FiO2 delivered, the oxygen flow rate, the alveolar–capillary membrane, the preoxygenation previously performed, the jaw luxation and the degree of hypoxaemia [16, 17]. In patients with obesity, a recent meta-analysis found that there might be no difference between HFNO and facemask preoxygenation in preventing oxygen desaturation during intubation [11].

A physiologic study [18] provided some explanations to the apparent discrepant results observed in the literature. When used as a method of preoxygenation, HFNO provides lower EtO2 than facemask preoxygenation [18]. Therefore, it seems that this method cannot replace preoxygenation using facemask or noninvasive ventilation [12]. Similarly, it cannot replace facemask ventilation, which allows a better ventilation of the patient than administration of oxygen only. However, it could be an interesting adjunct for apnoeic oxygenation, after the end of preoxygenation [16].

In the operating room, HFNO alone can be used in various settings: during the intubation procedure (for preoxygenation and/or apnoeic oxygenation) or during procedures without intubation (oro-pharyngeal surgeries, upper or lower endoscopy). When used in operating rooms, HFNO was often called “Trans nasal humidified rapid-insufflation ventilatory exchange (THRIVE)”.

In this setting, apnoeic oxygenation has been previously evaluated. A first study investigated the influence of HFNO alone on the duration of arterial oxygen saturation (SpO2) ≥ 95% in simulated difficult laryngoscopy in obese patients [19]. HFNO alone was associated with significant increases in SpO2 frequency and duration ≥ 95% and higher minimal SpO2 in prolonged laryngoscopies in obese patients. A randomized controlled trial [9], including 20 patients in each group, compared HFNO pre-oxygenation to facemask pre-oxygenation in patients undergoing emergency surgery, showing no difference in mean PaO2, PaCO2 or pH. However, this study was not powered to find a difference in complications related to intubation. In the setting of emergency surgery in patients also undergoing rapid sequence induction, a recent study [20] showed that in eighty adult patients fewer desaturation events were observed with HFNO alone for rapid sequence induction compared with facemask pre-oxygenation. Another randomized controlled trial [21] assessed the use of HFNO in both pre-oxygenation and apnoeic oxygenation in adults who were intubated following a non-RSI. Fifty patients were randomized to receive pre-oxygenation via a standard facemask or HFNO. No complications were observed in either group. HFNO produced a higher PaO2 after pre-oxygenation and safe PaO2 during intubation. Another preliminary study [22] found similar results. No previous study has simultaneously assessed facemask and HFNO for optimizing both preoxygenation and apnoeic oxygenation in a large multinational study.

Notably, we report that the EtCO2 within 2 min after intubation was similar in the facemask with HFNO group when compared with the facemask alone group. These results are in line with recent randomized controlled trials which does not support an additional ventilatory effect of HFNO [23, 24].

The present study has several strengths. First, we enrolled a large number of tracheal intubations (i.e., 450) over a 3 month period from three countries (USA, China and France). Another strength was the pragmatic broad spectrum of providers (senior anaesthesiologists, juniors, anaesthesiologist nurses, student anaesthesiologist nurses), of time of intubation (night and day shifts) and of level of emergency (scheduled or emergency surgeries), allowing an extrapolation of the results to all intubation providers and operating room settings. The positive results of the combination of standard face mask and apnoeic oxygenation were consistently found in the subgroup of patients with RSI. As the patients with RSI, by definition, usually do not get ventilated by face mask, this method could be even more interesting in this subgroup of patients.

Some limitations should also be mentioned. First, some data were lacking. However, they were clearly stated in all tables, and considered missing completely at random. Consequently, the complete case analysis was unbiased. Second, the design of the study was observational and not randomized. The EtO2 post intubation will depend on several factors including the condition of the patient, duration of apnea, number of attempts at intubation, mask ventilation from paralysis to tracheal intubation, which are difficult to compare in a before and after study. The level of proof in the present study is not as high as the level of proof of a randomized controlled trial. However, the assessment of a large sample size in a real-life setting, with a real-life control group, has also some strengths, and some authors suggest that large before–after studies can provide level of proof equal to RCTs [25, 26]. There is a well-known discrepancy between trials demonstrating efficacy (the intervention works in clinical trials under optimum conditions) and studies assessing effectiveness (the intervention works in the real world). Further studies on medico-economic aspects are needed to assess the relative cost-effectiveness of the implementation of HFNO for apnoeic oxygenation. Third, the main outcome, the lowest EtO2 within the 2 min after intubation, can be discussed. For comparable median duration of apnoea and assumed similar oxygen uptake in this randomised study, higher EtO2 after intubation suggests that facemask combined with HFNO stocks a higher amount of oxygen than facemask alone during preoxygenation. Moreover, this main outcome was used in recent studies in the field of preoxygenation [12, 27, 28].

## Conclusions

In summary, this study showed for the first time that combining facemask preoxygenation with HFNO for both preoxygenation and apnoeic oxygenation allowed improving EtO2 levels following intubation, compared to usual care with standard facemask preoxygenation. These results were consistent among patients without and with obesity and could have major implications in clinical practice, with the potential to change the first-intention intubation practices worldwide.

## Supplementary Information

**Additional file 1: Table S1.** Inclusion numbers by centre. **Fig. S1.** a. Face mask preoxygenation alone (standard) b. Combination of two methods: face mask preoxygenation (standard) associated to apneic oxygenation with high-flow nasal oxygen. **Fig. S2.** Desaturation (SpO2≤95%) rate during and within 2 min after intubation among patients with obesity in the face mask alone group and the face mask combined with HFNO group

## Data Availability

Research data and other material (e.g., study protocol and statistical analysis plan) will be made available to the scientific community, immediately on publication, with as few restrictions as possible. All requests should be submitted to the corresponding author who will review with the other investigators for consideration. A data use agreement will be required before the release of participant data and institutional review board approval as appropriate.

## Abbreviations

ASA: American Society of Anaesthesiology
BMI: Body mass index
CI: Confidence interval
EtCO2: End-tidal carbon dioxide concentration
EtO2: End-tidal oxygen concentration
FiO2: Fraction of inspired oxygen
HFNO: High-flow nasal oxygen
IQR: Interquartile range
PAO2: Oxygen alveolar partial pressure
SD: Standard deviation
SpO2: Peripheral oxygen saturation
