# Supplementary material for: Preoxygenation with standard facemask combining apnoeic oxygenation using high flow nasal cannula versuss standard facemask alone in patients with and without obesity: the OPTIMASK international study
Source: Ann Intensive Care. 2023 Apr 4;13:26. doi: 10.1186/s13613-023-01124-x (PMC10073359; doi:10.1186/s13613-023-01124-x)

Appendix

Preoxygenation with Standard Facemask Combined with Apnoeic Oxygenation High Flow Nasal Cannula versus Standard Facemask Alone in patients with and without obesity: the OPTIMASK international study

Table S1. Inclusion numbers by centre

	Overall (n=450)	Face mask alone (n = 233)	Face mask combined with HFNO (n = 217)
Boston (USA)	109 (24·2)	60 (25·8)	49 (22·6)
Montpellier (France)	123 (27·3)	62 (26·6)	61 (28·1)
Shanghai (China)	81 (18·0)	40 (17·2)	41 (18·9)
Zhengzhou (China)	137 (30·4)	71 (30·5)	66 (30·4)

Data are n/N (%).

Figure S1.a. Face mask preoxygenation alone (standard)

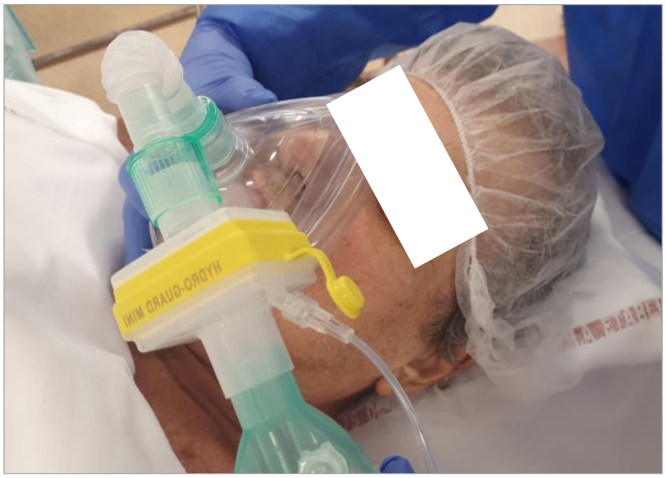

Figure S1.b. Combination of two methods: Face mask preoxygenation (standard) associated to apneic oxygenation with high-flow nasal oxygen

1. Preoxygenation with face mask
Before Intubation (patient awake)

+

2. Apneic oxygenation
(no breath patient)
(before); during and after intubation
(Continuous flow during the laryngoscopy)

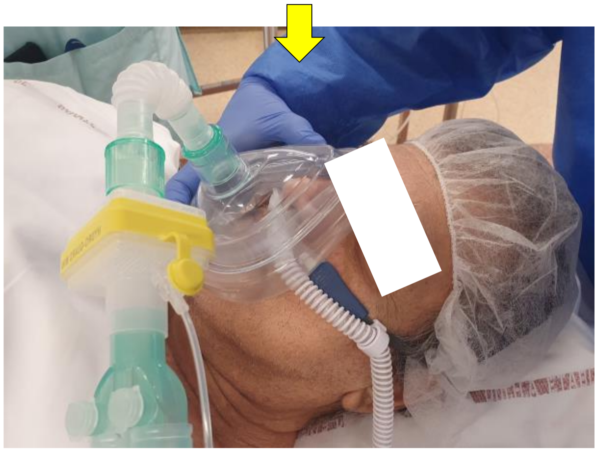

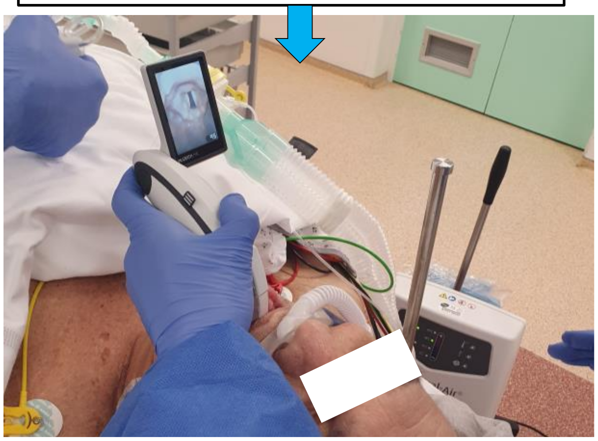

Figure S2. Desaturation (SpO2≤95%) rate during and within the two minutes after intubation among patients with obesity in the face mask alone group and the face mask combined with HFNO group

Abbreviations: SpO2, Peripheral oxygen saturation; HFNO, High-Flow Nasal cannula Oxygen

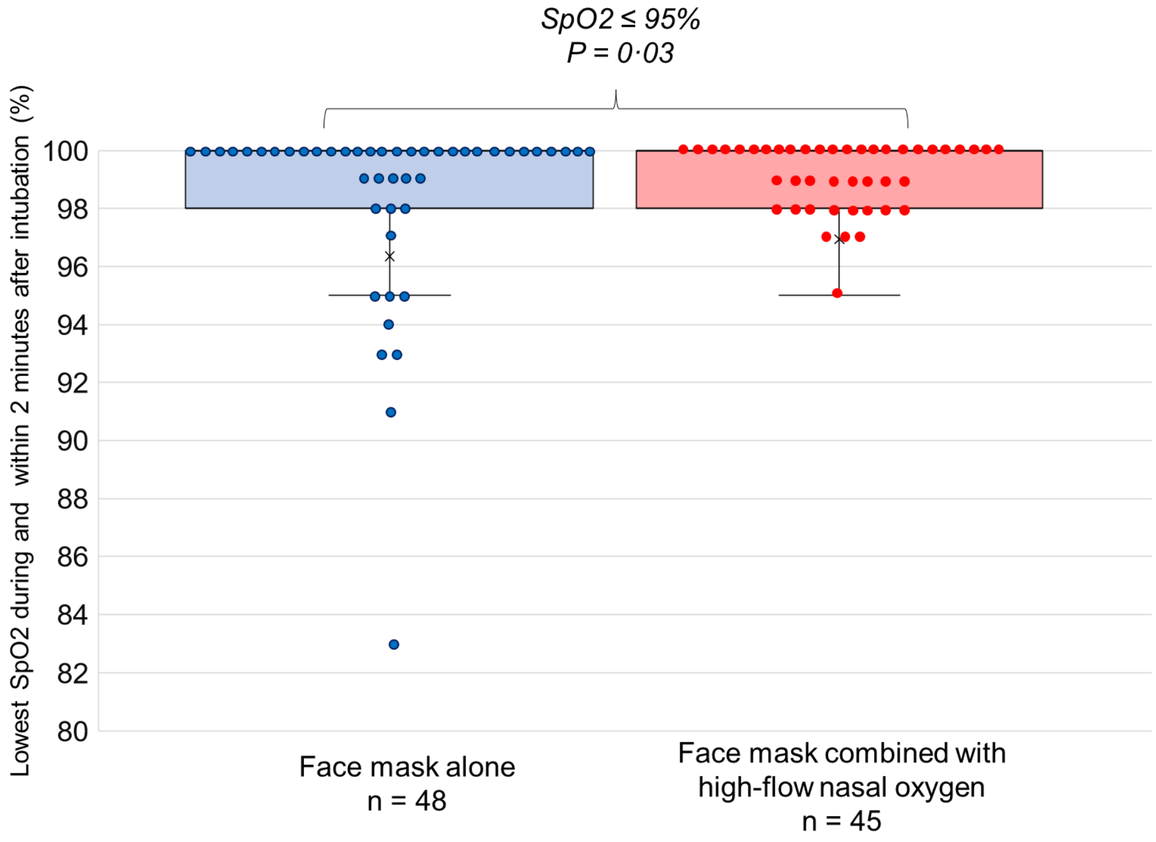

Supplement: Supplementary file 1 — Additional file 1: Table S1. Inclusion numbers by centre. Fig. S1. a. Face mask preoxygenation alone (standard) b. Combination of two methods: face mask preoxygenation (standard) associated to apneic oxygenation with high-flow nasal oxygen. Fig. S2. Desaturation (SpO2≤95%) rate during and within 2 min after intubation among patients with obesity in the face mask alone group and the face mask combined with HFNO group [file 13613_2023_1124_MOESM1_ESM.pdf]
